# Appearance of Pancreas Predictive of Cancer Presence: Utility of Computed Tomography Volumetry

**DOI:** 10.3390/cancers18111684

**Published:** 2026-05-22

**Authors:** Yuki Kawaji, Kentaro Yamao, Reiko Ashida, Mamoru Takenaka, Shunsuke Omoto, Ke Wan, Tomokazu Ishihara, Yuto Sugihara, Hiromu Morishita, Akiya Nakahata, Takahiro Shishimoto, Takashi Tamura, Yasunobu Yamashita, Masahiro Itonaga, Masayuki Kitano

**Affiliations:** 1 Second Department of Internal Medicine, Wakayama Medical University, 811-1 Kimiidera, Wakayama 641-0012, Wakayama, Japan; y-kawaji@wakayama-med.ac.jp (Y.K.); rashida@wakayama-med.ac.jp (R.A.); ishihara@wakayama-med.ac.jp (T.I.); s-yuto@wakayama-med.ac.jp (Y.S.); mrst@wakayama-med.ac.jp (H.M.); nakaaki@wakayama-med.ac.jp (A.N.); t-shishi@wakayama-med.ac.jp (T.S.); ttakashi@wakayama-med.ac.jp (T.T.); yasunobu@wakayama-med.ac.jp (Y.Y.); itonaga@wakayama-med.ac.jp (M.I.); 2 Department of Gastroenterology and Hepatology, Faculty of Medicine, Kindai University, 377-2, Sayama 589-8511, Osaka, Japan; yamaken_volvo@yahoo.co.jp (K.Y.); matakenaka@med.kindai.ac.jp (M.T.); s-omoto@med.kindai.ac.jp (S.O.); 3 Department of Gastroenterology and Hepatology, Nagoya University Graduate School of Medicine, 65 Tsuruma-cho, Showa-ku, Nagoya 466-8550, Aichi, Japan; 4 Clinical Study Support Center, Wakayama Medical University, 811-1 Kimiidera, Wakayama 641-0012, Wakayama, Japan; kwan@wakayama-med.ac.jp

**Keywords:** atrophy, pancreatic cancer, pancreatic volume, volumetry, high risk individuals

## Abstract

Background/Objectives: Pancreatic cancer (PC) should be diagnosed in its early stages. Therefore, it is necessary to identify high-risk individuals of PC. Methods: Between 2001 and 2017, 1542 PC cases were diagnosed at two tertial care institutions. Of these, 117 cases had undergone abdominal contrast-enhanced computed tomography (CE-CT) 1–10 years before PC diagnosis and were classified as the PC group. Meanwhile, 43,102 cases underwent abdominal CE-CT for close examination of non-pancreatic diseases in the same period, of which 1170 were randomly selected. Of these, 117 cases were matched to the PC group with the propensity score and designated the non-PC group. Pancreatic volumetry was performed using the 3D image analysis system for abdominal CE-CT in both groups and various measurements were compared. In PC group, CE-CT taken 1–10 years before the onset of PC was analyzed. Results: After propensity score matching, baseline characteristics did not significantly differ between the two groups. The whole pancreatic volume/body surface area (BSA) (*p* = 0.014), volume of main pancreatic duct (MPD) plus cystic lesion/BSA (*p* < 0.001), volume of pancreatic parenchyma/BSA (*p* = 0.002), ratio of cross-sectional areas (*p* = 0.033), and MPD diameter/BSA (*p* < 0.001) significantly differed between the two groups. In subgroup analysis of patients without cystic lesions, the whole pancreatic volume/BSA, volume of MPD/BSA, volume of pancreatic parenchyma/BSA, ratio of cross-sectional areas, and MPD diameter/BSA significantly differed between the two groups. Conclusions: Pancreatic volumetry could identify patients at high risk of PC.

## Simple Summary

Early diagnosis of pancreatic cancer (PC) is essential to improve prognosis, but remains challenging. Identifying high-risk individuals is therefore important. In this retrospective study, we evaluated whether pancreatic volumetric measurements obtained from contrast-enhanced CT scans before the diagnosis of PC could predict its future presence. We found that several quantitative imaging features, including small pancreatic parenchymal volume, large ductal and cystic lesions, and pancreatic duct dilation, were associated with a higher risk of PC. These findings were also consistent in patients without cystic lesions. Our results suggest that pancreatic volumetry may be a useful, non-invasive method for identifying high-risk individuals of PC. This approach may facilitate earlier detection and thereby improve the prognosis of PC.

## 1. Introduction

Pancreatic cancer (PC) is often diagnosed at an advanced stage and is associated with a poor prognosis; however, early detection improves clinical outcomes [1,2,3,4,5]. Therefore, identifying individuals at high risk of PC is essential.

Previous studies investigated the risk of PC presence using computed tomography (CT) imaging and identified factors such as main pancreatic duct (MPD) dilation, pancreatic cysts, and localized pancreatic atrophy as risk factors [6,7,8,9,10,11,12,13]. However, these assessments largely relied on subjective evaluations. We have conducted research with CT volumetry to objectively analyze imaging features associated with subsequent diagnosis of PC.

We previously reported the assessment of the pancreatic parenchyma prone to PC using pancreatic volumetry [14]. We revealed the following CT findings suggestive of underlying PC: (1) large volume of the MPD plus cystic lesions, (2) small volume of the pancreatic parenchyma, and (3) large MPD diameter. However, the previous study only had small sample numbers from Wakayama-Minami Radiology Clinic. Therefore, the present study incorporated a larger number of patients from two academic hospitals.

The aim of the present study was to perform pancreatic volumetry using abdominal contrast-enhanced CT (CE-CT) taken before the onset of PC in more patients than our previous study and to quantitatively evaluate CT findings associated with subsequent PC diagnosis.

## 2. Methods

### 2.1. Study Design

This retrospective, two-center study was performed at Wakayama Medical University and the Kindai University Faculty of Medicine. Radiological findings were compared between patients with pancreatic cancer (PC group) and those without pancreatic cancer (non-PC group). To minimize selection bias, patients in the non-PC group were selected using propensity score matching. The study was conducted in accordance with the Declaration of Helsinki and was approved by the ethics committees of Wakayama Medical University and Kindai University.

### 2.2. Patients

Medical records from Wakayama Medical University and Kindai University were retrospectively reviewed to identify patients with PC who had undergone abdominal CE-CT between 2001 and 2017. Patients were included in the PC group if they met the following criteria: (1) histologically confirmed PC or the presence of a pancreatic tumor suggestive of PC accompanied by evident metastases, (2) availability of a prior abdominal CE-CT scan that did not demonstrate PC, and (3) the interval between the CT examination before PC diagnosis and the diagnosis of PC ranged from 12 to 120 months (1–10 years). Definite PC was defined as a hypovascular pancreatic mass identified on abdominal CE-CT, while suspected PC was defined as a subtle or non-hypovascular mass.

For the non-PC group, we retrospectively reviewed medical records of patients who had undergone abdominal CE-CT for diseases unrelated to PC. CT examinations in the non-PC group were obtained during the same period as those in the PC group. The inclusion criteria for the non-PC group were as follows: (1) undergoing abdominal CE-CT for conditions other than pancreatic disease and (2) no evidence of PC. Subsequently, patients in the non-PC group were randomly selected, and the number of selected patients was set at ten times that of the PC group.

The following exclusion criteria were applied to both groups: (1) presence of a pancreatic solid tumor other than PC, (2) congenital pancreatic malformation, and (3) a history of pancreatectomy, (4) patients with imaging findings suggestive of acute pancreatitis or acute exacerbation of chronic pancreatitis at the time of CT acquisition.

### 2.3. Propensity Score Matching

To minimize selection bias, 1:1 propensity score matching was performed. Propensity scores were estimated by logistic regression analysis using age, sex, body mass index (BMI), and body surface area (BSA) as covariates. Matching was subsequently carried out using the nearest-neighbor method with a caliper coefficient of 1.95.

### 2.4. CT Scanning Protocol

CT examinations were performed using multiple scanner models during the study period; in Wakayama Medical University, High Speed Advantage (GE Medical Systems, Milwaukee, WI, USA) from 2001 to 2003, Aquilion 16 (Canon Medical Systems, Otawara, Japan) from 2004 to 2012, LightSpeed VCT (GE Medical Systems, Milwaukee, WI, USA) from 2006 to 2016, and Aquilion ONE (Canon Medical Systems, Otawara, Japan) from 2013 to 2016. Image reconstruction was performed using a standard kernel for GE scanners and FC13 for Canon scanners. CE-CT was performed with intravenous administration of iodinated contrast material (Iomeron^®^ 350, Bracco, Italy) at a dose of 600 mgI/kg. In Kindai University, CT examinations were performed using Aquilion (Canon Medical Systems, Otawara, Japan) from 2001 to 2010 and Aquilion PRIME (Canon Medical Systems, Otawara, Japan) from 2011 to 2016. Image reconstruction was performed using FC01 for Aquilion and FL03 for Aquilion PRIME. CE-CT was performed with intravenous administration of iodinated contrast material (Iopamiron^®^ 300 or 370, Bayer, Osaka, Japan) at a dose of 600 mgI/kg.

### 2.5. Pancreatic Volumetry

CT images were analyzed using SYNAPSE VINCENT version 6.8.0037 (Fujifilm Medical Co., Ltd.) image analysis software. In the PC group, pancreatic volumetry parameters were measured using the last CT scan obtained before the diagnosis of PC. Pancreatic volumetry was generally performed using portal phase images. Two experienced gastroenterologists specializing in pancreatology (Y.K. and R.A.) evaluated the CT images and determined the measurement lines. First, one gastroenterologist performed the volumetric measurements and defined the measurement lines. Then, a second gastroenterologist reviewed the measurements and modified the measurement lines when necessary. If the measurement lines were difficult to determine, both reviewers evaluated the images together and reached a consensus. They were blinded to the whole patient’s information including final diagnosis during measurement of the imaging parameters.

**Figure 1:**
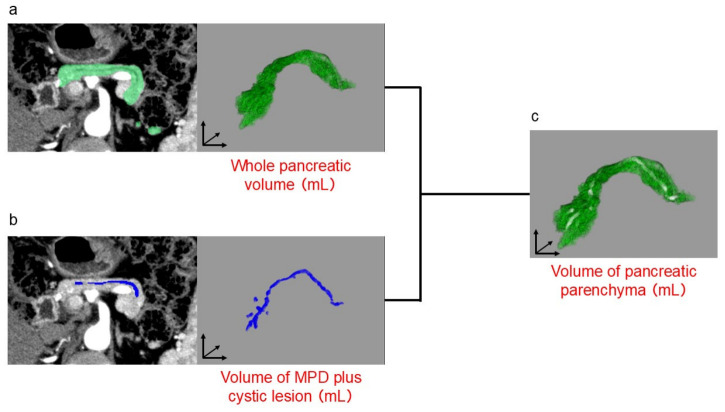
Pancreatic volumetry: 3D analysis. (**a**) Tracing the whole pancreas and measurement of the whole pancreatic volume (green color). (**b**) Tracing of the MPD and cystic lesions and measurement of the volume of the MPD plus cystic lesions (blue color). (**c**) Subtraction of the volume of the MPD plus cystic lesions from the whole pancreatic volume to calculate the volume of the pancreatic parenchyma (green color excluding white). MPD, main pancreatic duct.

In the 3D analysis (Figure 1), pancreatic volumetry was performed as follows: (1) the whole pancreas was traced to measure the whole pancreatic volume; (2) the MPD and cystic lesions were traced to measure their combined volume; and (3) the volume of pancreatic parenchymal was calculated by subtracting the volume of the MPD and cystic lesions from the whole pancreatic volume.

**Figure 2:**
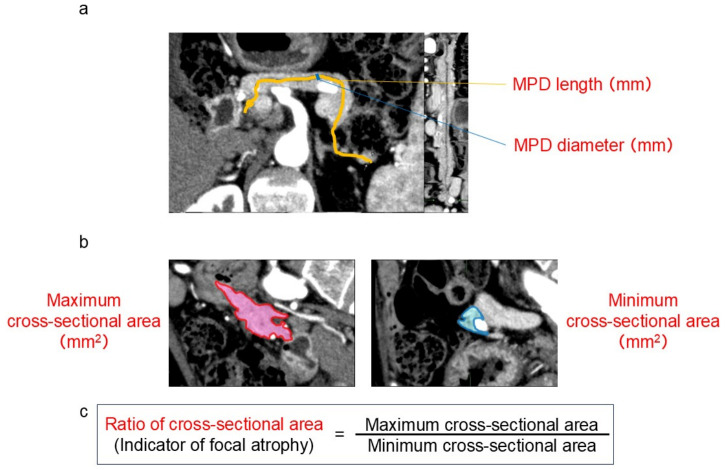
Pancreatic volumetry: CPR analysis. (**a**) Creation of CPR along the MPD and measurement of the length (yellow color) and maximum diameter (blue color) of the MPD. (**b**) Measurement of the maximum (red color) and minimum (blue color) cross-sectional areas of the pancreatic parenchyma vertical to the MPD using CPR. (**c**) Calculation of the ratio of the cross-sectional areas, defined as the maximum cross-sectional area divided by the minimum cross-sectional area, which is an indicator of focal atrophy of the pancreatic parenchyma. CPR, curved multiplanar reconstruction; MPD, main pancreatic duct.

In the curved multiplanar reconstruction (CPR) analysis (Figure 2), the following measurements were obtained: (1) CPR images were generated along the MPD to measure the MPD length and maximum diameter, and (2) the maximum and minimum cross-sectional areas of the pancreatic parenchyma perpendicular to the MPD were measured using CPR.

To investigate factors associated with future pancreatic carcinogenesis, the following pancreatic volumetry parameters were evaluated (Figure 1 and Figure 2): whole pancreatic volume, volume of the MPD plus cystic lesions, volume of the pancreatic parenchyma, maximum cross-sectional area of the pancreas, minimum cross-sectional area of the pancreas, ratio of the cross-sectional areas, MPD length, and MPD diameter.

The volume of the pancreatic parenchyma was determined by subtracting the volume of the MPD plus cystic lesions from the whole pancreatic volume. The minimum cross-sectional area of the pancreas was measured in regions excluding the terminal portions of the pancreatic head and tail. The ratio of the cross-sectional areas was calculated as the maximum cross-sectional area divided by the minimum cross-sectional area and was used to represent focal pancreatic parenchymal atrophy. MPD length referred to the distance from the margin of the pancreatic head to the end of the pancreatic tail along the MPD. MPD diameter was defined as the largest diameter of the MPD identified on imaging. The time to PC diagnosis was calculated as the period between the CT scan obtained before PC diagnosis and the CT scan at the time of the initial diagnosis of PC.

### 2.6. Correlation Between the Volume of the Pancreatic Parenchyma and BSA

Previous studies have demonstrated a correlation between pancreatic volume and BSA [14,15,16,17,18]. Therefore, in the non-PC group, the relationship between the volume of the pancreatic parenchyma and BSA was assessed using Pearson’s correlation coefficient. To adjust for differences in body size, the volume of the pancreatic parenchyma and other pancreatic volumetry parameters were normalized by dividing each value by the BSA for subsequent analyses.

### 2.7. Statistical Analysis

Each measured parameter on pancreatic volumetry was compared between the PC and non-PC groups. Subsequently, in patients without cystic lesions, subgroup analysis comparing the PC and non-PC groups was performed. Continuous variables were presented as median and IQR, and compared between the two groups using Student’s *t* test. Categorical variables were presented as frequency and percentage, and compared between the two groups using the Chi-squared or Fisher’s exact tests. All reported *p* values are two-sided, and *p* values < 0.05 were considered statistically significant. Statistical analyses were performed using JMPJMP Pro version 16 (SAS Institute Inc., Cary, NC, USA).

## 3. Results

### 3.1. Patients

**Figure 3:**
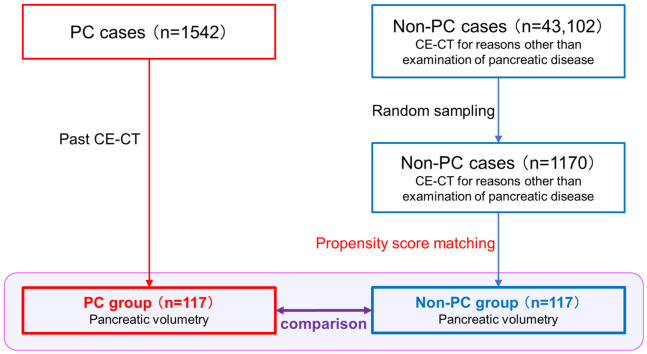
Patient selection. PC, pancreatic cancer; CE-CT, contrast-enhanced computed tomography.

Patient selection is shown in Figure 3. A total of 1542 patients with PC were admitted to two institutions between 2001 and 2017. Among these, 117 patients who had undergone CE-CT and satisfied the inclusion criteria were assigned to the PC group. The past CE-CT scans of patients in the PC group were performed between 2001 and 2016. Between 2010 and 2016, a total of 43,102 patients underwent CE-CT for indications unrelated to pancreatic disease. Among them, 1170 patients, corresponding to ten times the number of patients in the PC group, were randomly selected based on the predefined inclusion and exclusion criteria. Patient characteristics are summarized in Table 1. BSA was significantly different between the PC and non-PC cases (1.68 vs. 1.58; *p* < 0.001).

### 3.2. Patient Characteristics After Propensity Score Matching

Using the factors of age, sex, BMI, and BSA, we calculated the propensity scores and matched 117 patients in the non-PC group with 117 patients in the PC group (Figure 3). After selection using 1-to-1 propensity score matching, baseline characteristics did not significantly differ between the PC and non-PC groups including BSA (PC group vs. non-PC group: 1.68 vs. 1.68; *p* = 0.321; Table 2).

### 3.3. Correlation of Pancreatic Volume with BSA

In the non-PC group, the volume of the pancreatic parenchyma showed a significant correlation with BSA (r = 0.41, *p* < 0.001; Figure S1).

### 3.4. Comparisons Between the PC and Non-PC Groups

Various parameters were compared between the PC and non-PC groups after propensity score matching. The whole pancreatic volume/BSA (PC group vs. non-PC group: 35.2 vs. 39.4 mL/m^2^, *p* = 0.014), volume of the MPD plus cystic lesions/BSA (1.3 vs. 0.6 mL/m^2^, *p* < 0.001), volume of pancreatic parenchyma/BSA (33.7 vs. 38.5 mL/m^2^, *p* = 0.002), ratio of cross-sectional areas (3.7 vs. 3.3, *p* = 0.033), and MPD diameter/BSA (1.4 vs. 1.0 mm/m^2^, *p* < 0.001) significantly differed between the two groups (Table 3). The median thickness of CT slices did not significantly differ between the two groups (5.0 vs. 5.0 mm, *p* = 0.160). The median time to PC diagnosis in the PC group was 2.0 years (interquartile range, 1.3–3.9 years).

### 3.5. Comparisons Between the PC and Non-PC Groups in Subgroup Analysis of Patients Without Cystic Lesions

In subgroup analysis of patients without cystic lesions, the whole pancreatic volume/BSA (PC group vs. non-PC group: 34 vs. 40.6 mL/m^2^, *p* = 0.010), volume of MPD/BSA (0.9 vs. 0.4 mL/m^2^, *p* < 0.001), volume of pancreatic parenchyma/BSA (33.6 vs. 40 mL/m^2^, *p* = 0.005), ratio of cross-sectional areas (3.9 vs. 3.2, *p* = 0.048), and MPD diameter/BSA (1.3 vs. 1.0 mm/m^2^, *p* = 0.001) significantly differed between the two groups (Table 4).

## 4. Discussion

The present study analyzed pancreatic volumetry measured on abdominal CE-CT taken before the onset of PC to predict the future occurrence of PC. We identified several objective and quantitative features of the pancreas that increase the possibility of PC presence. The whole pancreatic volume/BSA (PC group vs. non-PC group: 35.2 vs. 39.4 mL/m^2^, *p* = 0.014), volume of MPD plus cystic lesions/BSA (1.3 vs. 0.6 mL/m^2^, *p* < 0.001), volume of pancreatic parenchyma/BSA (33.7 vs. 38.5 mL/m^2^, *p* = 0.002), ratio of cross-sectional areas (3.7 vs. 3.3, *p* = 0.033), and MPD diameter/BSA (1.4 vs. 1.0 mm/m^2^, *p* < 0.001) significantly differed between the two groups (Table 3).

Previous studies reported that cystic lesions of the pancreas and dilatation of the MPD are observed on CT scans before diagnosis of PC [6,7,8,10,11,19]. A study mentioned that focal pancreatic abnormalities, including focal atrophy, faint enhancement, and MPD change, are seen before diagnosis of PC [9,10,12]. These reports showed findings similar to those of our study. However, these findings were based solely on subjective assessment. In contrast, the present study employed objective methods to evaluate CT findings. Furthermore, we demonstrated that whole pancreatic atrophy, in addition to focal pancreatic atrophy, was related to future occurrence of PC. This was possible only because of objective and quantitative volumetry.

Our previous study revealed that the volume of the MPD plus any cystic lesion/BSA ≥ 0.53 mL/m^2^, a volume of pancreatic parenchyma/BSA < 27.0 mL/m^2^, and a MPD diameter/BSA ≥ 1.0 mm/m^2^ are significant risk factors for PC [14,20,21,22,23,24,25,26]. The present study also identified these as risk factors. However, the previous study was performed in a small institution (Wakayama-Minami Radiology Clinic) and had a small sample size (only 15 PC patients). The present study incorporating a larger number of patients with PC (117 PC patients) confirmed those results. Based on the above, there was no doubt that atrophy of the pancreatic parenchyma, pancreatic cysts, and MPD dilation are critical risk findings for presence of PC, and pancreatic volumetry is considered highly valuable to assess these findings.

In addition, the present study assessed focal atrophy of the pancreatic parenchyma using a quantitative method and revealed that this finding indicates a high risk of PC presence. In recent years, focal atrophy of the pancreatic parenchyma was reported to be an important finding in early PC [12,13,27]. However, the problem is that the definition of focal pancreatic atrophy has not been established. In the present study, we defined the ratio of the cross-sectional areas as an indicator of pancreatic focal atrophy. As mentioned above, the ratio of the cross-sectional areas was significantly larger in the PC group than in the non-PC group (3.7 vs. 3.3, *p* = 0.033). This result indicates that pancreatic focal atrophy was more frequently seen in the PC group. Therefore, this evaluation method must be highly innovative and prove the utility of pancreatic volumetry.

In subgroup analysis of patients without cystic lesions, the whole pancreatic volume/BSA (*p* = 0.01), volume of MPD/BSA (*p* < 0.0001), volume of pancreatic parenchyma/BSA (*p* = 0.005), ratio of cross-sectional areas (*p* = 0.048), and MPD diameter/BSA (*p* = 0.001; Table 4) significantly differed between the two groups. These results mean that pancreatic volumetry can detect individuals at high risk of PC presence other than those with pancreatic cysts or intraductal papillary mucinous neoplasm (IPMN) [28,29]. Even in individuals without pancreatic cysts or IPMN, an increased MPD diameter or volume, pancreatic parenchymal atrophy, and focal pancreatic atrophy are risk factors for PC [13,20,21,22,30].

Although further validation is required, these findings may have potential implications for clinical practice. Individuals incidentally found to have significant CT findings such as pancreatic parenchymal atrophy, MPD dilation, or an elevated ratio of cross-sectional areas may benefit from closer surveillance, such as periodic CT or endoscopic ultrasound follow-up, with the aim of detecting PC at an earlier and potentially resectable stage. However, the optimal screening interval and the cost-effectiveness of such an approach remain to be established in future prospective studies.

While pancreatic volumetry using commercially available 3D image analysis software is feasible in specialized centers, its use in non-specialized settings may be limited by the availability of specific software and the expertise required for accurate measurements. Standardization of the volumetric protocol and training of operators will be essential before widespread clinical adoption.

It is unclear how early changes in the pancreatic parenchyma can be detected on CT before the development of PC. Most PC arise from microscopic precursor lesions known as pancreatic intraepithelial neoplasia (PanIN) through a stepwise progression process [31]. Yachida et al. conducted a quantitative analysis of the timing of genetic evolution in PC and estimated that, on average, 11.7 years elapse from PanIN initiation to the emergence of the parental clone, followed by 6.8 years to the development of the index lesion, and a further 2.7 years until metastatic dissemination and patient death [32]. These findings suggest that relevant changes in the pancreatic parenchyma may be detectable well before the development of invasive carcinoma. A multicenter study investigating CT findings prior to the diagnosis of PC in 320 patients reported that focal parenchymal atrophy was the earliest detectable imaging abnormality before diagnosis [33]. This finding was observed, on average, 2.7 years before the diagnosis of PC. Among 85 patients with focal parenchymal atrophy, two cases showed this finding 7–10 years prior to PC diagnosis. Notably, no cases exhibited CT abnormalities more than 10 years before diagnosis. Based on these findings, we considered that our observation period of 1–10 years before the diagnosis of PC was appropriate.

We recognize that chronic pancreatitis may show imaging features similar to PC, particularly in terms of parenchymal atrophy [25,26,27]. In the present study, two cases with chronic pancreatitis were included in the PC group, and five cases were included in the non-PC group. These seven cases were diagnosed as chronic pancreatitis based on the presence of calcifications within the pancreatic parenchyma on CT. However, due to the small number of patients with chronic pancreatitis, it was difficult to assess whether CT volumetry can differentiate between PC and chronic pancreatitis. Further studies are needed to evaluate the utility of CT volumetry in patients with chronic pancreatitis.

The present study has several limitations. First, it was not a prospective study aimed at cancer surveillance in high-risk individuals. It was a retrospective analysis examining pre-PC diagnosis CT characteristics in patients with incidentally identified PC. Therefore, this retrospective study is subject to selection bias, as the PC group comprised patients who had incidentally undergone CT imaging prior to their diagnosis, and may not represent the broader population of individuals at risk for PC. The purpose of this study was to identify characteristics of the pancreatic parenchyma that may lead to PC. Therefore, we performed pancreatic volumetry of abdominal CE-CT scans acquired 12–120 months (1–10 years) before diagnosis of PC. Although patients with acute pancreatitis or acute exacerbation of chronic pancreatitis were excluded, subclinical or unrecognized chronic pancreatitis could not be completely ruled out. In addition, the history of prior acute pancreatitis was not fully available in this study. Therefore, some of the CT findings identified in this study may partially reflect underlying or prior pancreatitis, which is a known risk factor for PC [34]. Additional research through a large prospective multicenter study is required to validate the findings of this study. Second, the CT slices were relatively thick. The median CT slice thickness was 5 mm in both groups. The slice thickness may affect accurate measurements during pancreatic volumetry. Therefore, it would be ideal to use thin CT slices with a thickness of 1–2 mm for pancreatic volumetry in a future study. Third, the study period was long, and since the study was conducted at two facilities, the CT equipment and imaging conditions were not standardized. Furthermore, the subgroup analysis of patients without cystic lesions included only 48 patients in the PC group, which may limit the statistical power of those findings. These results should therefore be interpreted with caution and require validation in larger cohorts.

## 5. Conclusions

In conclusion, several pancreatic volumetry findings on CE-CT obtained before diagnosis of PC can predict future PC presence. A large volume of the MPD plus cystic lesions, a dilated MPD, and whole or focal parenchymal atrophy were identified as risk factors for PC. Pancreatic volumetry using CE-CT can identify patients at high risk of PC presence.

## Figures and Tables

**Table 1:** Patient characteristics before matching.

	PC Group (*n* = 117)	Non-PC Cases (*n* = 1170)	*p*
Age * (years)	71 (64–75)	69 (62–77)	0.125
Sex (male)	81 (69.2)	712 (60.9)	0.076
BMI *	21.7 (20.0–25.5)	22.5 (20.0–25.0)	0.761
BSA *	1.68 (1.52–1.78)	1.58 (1.45–1.72)	<0.001

Values are expressed as the median (IQR) or n (%). PC, pancreatic cancer; BMI, body mass index; BSA, body surface area; IQR, interquartile range. * Values at the time of the pre-PC diagnosis CT scan in the PC group.

**Table 2:** Patient characteristics after matching.

	PC Group (*n* = 117)	Non-PC Group (*n* = 117)	*p*
Age * (years)	71 (64–75)	70 (62–76.5)	0.229
Sex (male)	81 (69.2)	84 (71.8)	0.667
BMI *	21.7 (20.0–25.5)	23.0 (20.3–25.4)	0.326
BSA *	1.68 (1.52–1.78)	1.68 (1.54–1.81)	0.321

Values are expressed as the median (IQR) or n (%). PC, pancreatic cancer; BMI, body mass index; BSA, body surface area; IQR, interquartile range. * Values at the time of the pre-PC diagnosis CT scan in the PC group.

**Table 3:** Comparison of the PC and non-PC groups.

	PC Group (*n* = 117)	Non-PC Group (*n* = 117)	95% CI	*P*
Whole pancreatic volume/BSA (mL/m^2^)	35.2 (29.0–44.1)	39.4 (32.4–48.3)	−6.55 to −0.76	0.014
Volume of MPD plus cystic lesions/BSA (mL/m^2^)	1.3 (0.8–1.9)	0.6 (0.4–1.1)	0.60 to 1.11	<0.001
Volume of pancreatic parenchyma/BSA (mL/m^2^)	33.7 (27.8–41.3)	38.5 (30.6–47.1)	−7.40 to −1.61	0.002
Maximum cross-sectional area/BSA (mm^2^/m^2^)	505.3 (382.7–621.6)	477.5 (376.6–615.0)	−35.65 to 70.27	0.520
Minimum cross-sectional area/BSA (mm^2^/m^2^)	130.7 (93.0–182.7)	147.7 (105.8–200.5)	−27.12 to 9.74	0.350
Ratio of cross-sectional areas	3.7 (2.8–5.2)	3.3 (2.6–4.7)	0.05 to 1.11	0.033
MPD length/BSA (mm/m^2^)	106.4 (93.6–119.4)	104.0 (96.8–114.9)	−3.71 to 4.74	0.810
MPD diameter/BSA (mm/m^2^)	1.4 (1.1–2.0)	1.0 (0.8–1.4)	0.35 to 0.75	<0.001

Values are expressed as the median (IQR). PC, pancreatic cancer; CI, confidence interval; BSA, body surface area; MPD, main pancreatic duct; IQR, interquartile range.

**Table 4:** Comparison of the PC and non-PC groups in subgroup analysis of patients without cystic lesions.

	PC Group (*n* = 48)	Non-PC Group (*n* = 65)	95% CI	*P*
Whole pancreatic volume/BSA (mL/m^2^)	34 (28.2–44.1)	40.6 (33.6–49.7)	−10.04 to −1.41	0.010
Volume of MPD/BSA (mL/m^2^)	0.9 (0.6–1.5)	0.4 (0.3–0.7)	0.31 to 0.74	<0.001
Volume of pancreatic parenchyma/BSA (mL/m^2^)	33.6 (26.9–43.3)	40 (32.6–48.9)	−10.59 to −1.92	0.005
Maximum cross-sectional area/BSA (mm^2^/m^2^)	496.7 (339.4–604.6)	460.7 (377.7–631.4)	−83.78 to 79.96	0.963
Minimum cross-sectional area/BSA (mm^2^/m^2^)	128.5 (92.7–184.5)	161.6 (112–212)	−53.34 to 0.43	0.054
Ratio of cross-sectional areas	3.9 (2.6–5.4)	3.2 (2.5–4.7)	0.01 to 1.66	0.048
MPD length/BSA (mm/m^2^)	105 (92–116.5)	104.2 (97.8–116.8)	−8.93 to 3.40	0.376
MPD diameter/BSA (mm/m^2^)	1.3 (1.0–1.6)	1.0 (0.7–1.3)	0.21 to 0.75	0.001

Values are expressed as the median (IQR). PC, pancreatic cancer; CI, confidence interval; BSA, body surface area; MPD, main pancreatic duct; IQR, interquartile range.

## Data Availability

The data presented in this study are available on request from the corresponding author. The data are not publicly available due to privacy issues.

## Supplementary Materials

The following supporting information can be downloaded at: https://www.mdpi.com/article/10.3390/cancers18111684/s1. Figure S1. Correlation between the volume of the pancreatic parenchyma and BSA in the non-PC group. There was a significant correlation between the volume of the pancreatic parenchyma and BSA in the non-PC group. BSA, body surface area; PC, pancreatic cancer.

## Abbreviations

The following abbreviations are used in this manuscript:

PC	pancreatic cancer
CE-CT	contrast-enhanced computed tomography
BSA	body surface area
MPD	main pancreatic duct
BMI	body mass index
CPR	curved multiplanar reconstruction
IQR	interquartile range
IPMN	intraductal papillary mucinous neoplasm
