# Supplementary material for: Appearance of Pancreas Predictive of Cancer Presence: Utility of Computed Tomography Volumetry
Source: Cancers (Basel). 2026 May 22;18(11):1684. doi: 10.3390/cancers18111684 (PMC13256071; doi:10.3390/cancers18111684)
Supplement: Supplementary file 1 [file cancers-18-01684-s001.zip › cancers-4252300-supplementary/cancers-4252300-supplementary.pdf]

cancers

MDPI

Supplementary

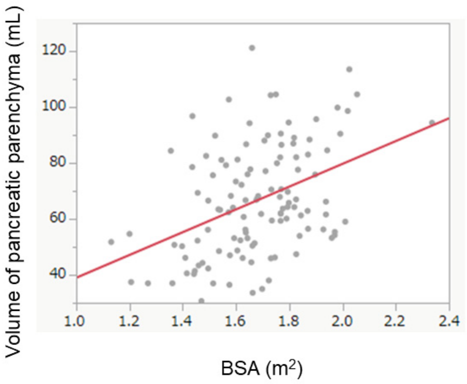

$$\text{Volume of pancreatic parenchyma (mL)} = -1.7 + 40.7 \times \text{BSA (m}^2\text{)}$$

$$(r = 0.41, P<0.0001)$$

Figure S1. Correlation between the volume of the pancreatic parenchyma and BSA in the non-PC group. There was a significant correlation between the volume of the pancreatic parenchyma and BSA in the non-PC group. BSA, body surface area; PC, pancreatic cancer.
